# Trends and Challenges for the Clinical Adoption of Fluorescence-Guided Surgery

**DOI:** 10.2967/jnumed.119.226795

**Published:** 2019-06

**Authors:** Jonathan T.C. Liu, Nader Sanai

**Affiliations:** 1 Department of Mechanical Engineering, University of Washington, Seattle, Washington; 2 Department of Pathology, University of Washington School of Medicine, Seattle, Washington; and; 3 Department of Neurological Surgery, Barrow Neurological Institute, Phoenix, Arizona

See the associated article on page 758.

Optical imaging with the unassisted eye, along with palpation, are the primary guides for surgical oncologists. Together with the subjective judgments of the surgeon, these sensory cues will continue to serve as the mainstay for tumor resection procedures. However, there is clearly a need for adjunct technologies to enable greater resection accuracy by providing greater tumor-detection sensitivity, higher spatial resolution, or increased quantitation and reproducibility. Advanced radiographic approaches such as CT and MRI are now routinely used to guide procedures that demand high precision, such as neurosurgical resections, either through preoperative imaging in conjunction with real-time navigation techniques, or through intraoperative imaging (1). However, the applicability of these techniques is limited due to the high cost and complexity of such radiographic approaches, as well as their limited resolution and sensitivity, especially for guiding operative decisions at the surgical margins where tumor burden is low or spatially disseminated. Here, the value of optical imaging is highest, for although the ability of light to penetrate deeply within tissues is poor, the exquisite sensitivity and spatial resolution that can be achieved at superficial depths (e.g., near the final resection margins) is of significant utility in the surgical armamentarium.

The recent early-stage clinical feasibility study by van Keulen et al. in this issue of *The Journal of Nuclear Medicine* (2) provides concrete examples of how fluorescence-guided surgery (FGS) can affect surgical decision making. The first and most obvious example is that of visualizing residual tumor at the base (“deep margin”) of a resection cavity after the surgeon has determined that resection is complete based on conventional means (i.e., visual and tactile feedback). Another example is identifying an unanticipated secondary lesion that is missed during standard visual inspection.

A confounding factor alluded to by van Keulen et al. (2) is the organ-specific definition of an acceptable margin of benign tissue that must exist between the excised tumor and the surgical margin surface (i.e., the “inked margin”). Here, one must recognize that current criteria for margins have evolved, in part, to compensate for the limitations of conventional postoperative histology, in which small numbers of thin tissue sections are imaged in the vertical (depthwise) direction, often at intervals of several millimeters (i.e., bread-loafing). The ability to comprehensively image the entire surgical margin surface with FGS should therefore motivate new bespoke criteria for surgical completeness. Such criteria will invariably be optimized and tailored over time to reflect our evolving understanding of the underlying biology and spatial characteristics of a specific disease, and as clinical studies reveal outcomes benefits (3). Central to this analysis is also an understanding of the targeting behavior of the specific fluorescent contrast agent being used.

Just as surgical-margin criteria cannot not be generalized across disparate diseases, a diverse array of FGS technologies have been developed to address specific clinical applications. As described in van Keulen et al. (2), low-resolution fluorescence surgical microscopy is the most-popular FGS approach in which numerous imaging platforms are now available (4). These systems provide the advantage of a wide-area view, often of the entire surgical field, but are limited in terms of detection sensitivity and the subjective nature of the imaging readout (5). Portable spectroscopy devices and high-resolution imaging probes sacrifice field of view but provide greater detection sensitivity and quantitative detection of tumor-specific contrast agents at localized tissue regions (5). In the case of high-resolution in vivo microscopes, microarchitectural details that approach the gold-standard of histopathology may be visualized in real time, both with and without the aid of exogenous agents (5). Ultimately, a combination of low-resolution (wide-area) and localized high-resolution detection techniques may be of value for many resection procedures. For example, bulk tumor and noncritical regions could be resected more aggressively under wide-area FGS, whereas regions that are vital for cosmetic or functional purposes could be resected with greater precision using localized probing techniques. Finally, there is an important role for “closed-field” ex vivo imaging technologies for FGS, which, unlike the aforementioned in vivo imaging approaches, allows for greater control over optical parameters such as illumination intensity and geometry, along with reduced interference from ambient light background, all of which ultimately enables more-accurate and quantitative visualization of tissue morphology and fluorescence contrast. Downsides include lengthened procedure times and potential degradation of image contrast and tissue quality after patient excision. A recent study by the same group led by Dr. Eben Rosenthal has attempted to compare several in vivo and ex vivo FGS platforms (6).

As FGS approaches achieve varying levels of maturity, improvements in image processing and analytics will likely play an outsized role in their success and impact. Initial efforts have focused on improving the robustness of signal acquisition in the presence of confounding factors such as ambient light contamination as well as misleading sources of image contrast such as changes in tissue-optical properties, variations in signal due to tissue geometry (i.e., working distance and angle of incidence), and the nonspecific accumulation of contrast agents due to passive mechanisms. Methods for mitigating these issues have involved creative combinations of hardware and image-processing methods, such as multispectral detection (7) and ratiometric “paired-agent” methods, in which a nonspecific control agent is simultaneously imaged with a targeted agent to provide a means to normalize for the misleading sources of contrast listed above (8). Future efforts in computational analysis and machine learning are needed to assist with clinical interpretation of FGS data. For example, van Keulen et al. (2) observe differences in both the mean fluorescence intensity and the spatial heterogeneity of FGS images obtained from benign and malignant tissue types. Automated segmentation and classification algorithms, ideally trained and validated through outcomes-based studies, can assist with subtle pattern-recognition tasks but will face challenges similar to others striving to implement artificial intelligence in health care (9).

Clinical validation and adoption are the final measures of success for FGS and other innovative approaches in medicine. Early successes should pave the way for accelerated translation of subsequent technologies. As a benchmark example, the phase 3 clinical study on the use of 5-ALA for FGS of high-grade gliomas was published by Stummer et al. in 2006 (10) and led to its regulatory approval in the European Union in 2007. However, subsequent Food and Drug Administration approval in the United States did not occur until a decade later in 2017. Although regulatory approval represents a real and complex challenge, adequate reimbursement of FGS techniques is of equal, if not greater, concern for broad clinical adoption by all but the most academically motivated institutions. Here, translational researchers and commercial entities are challenged to develop realistic financial models to demonstrate a compelling value proposition to payers. Such models should extend beyond the immediate financial benefits of reduced call-back surgeries for patients with positive margins (e.g., breast cancer lumpectomy) and include a long-term analysis of patient outcomes such as progression to advanced metastatic disease, which results in exponential increases in the cost of care, as well as the financial implications of the side effects caused by overtreatment (e.g., neurologic morbidity in the case of brain tumor resections). The article by van Keulen et al. (2) refers to the concept of clinically significant changes brought on by FGS. Ultimately, reimbursement strategies will require careful and precise definition of these clinically significant changes, along with the attendant benefits and risks to the patient, as viewed through the lens of the economics of care.

## DISCLOSURE

Dr. Jonathan Liu is a cofounder and shareholder of LightSpeed Microscopy Inc., which has licensed intellectual property generated by Dr. Liu’s laboratory at the University of Washington. No other potential conflict of interest relevant to this article was reported.
